# The relationship between retinal vessel calibre and knee cartilage and BMLs

**DOI:** 10.1186/1471-2474-13-255

**Published:** 2012-12-20

**Authors:** Miranda L Davies-Tuck, Ryo Kawasaki, Anita E Wluka, Tien Y Wong, Lauren Hodgson, Dallas R English, Graham G Giles, Flavia Cicuttini

**Affiliations:** 1 Department of Epidemiology and Preventive Medicine, Monash University, Central and Eastern Clinical School, Alfred Hospital, Melbourne, VIC, 3004, Australia; 2 Department of Ophthalmology, Centre for Eye Research Australia, Royal Victorian Eye and Ear Hospital, University of Melbourne, East Melbourne, VIC, 3002, Australia; 3 Baker Heart Research Institute, Melbourne, Australia; 4 Singapore Eye Research Institute, National University of Singapore, Singapore, Singapore; 5 Centre for Molecular, Environmental, Genetic and Analytic Epidemiology, School of Population Health, The University of Melbourne, Carlton, VIC, 3053, Australia; 6 Cancer Epidemiology Centre, The Cancer Council of Victoria, Carlton, VIC, 3053, Australia

## Abstract

**Background:**

Whether the increase in vascular disease prevalence and mortality in OA populations is a result of co-occurrence of cardiovascular disease and OA, which are both common in the older population, is due to OA treatments or to the common association with reduced physical activity and/or obesity is unclear. One way to explore this non-invasively is to examine the cross-sectional relationship between changes in retinal microvasculature, which have been shown to be markers of generalized vascular pathology, and knee structural changes in an asymptomatic community-based population.

**Methods:**

A community sample of 289 (61% women) aged 50–79 years with no knee symptoms underwent magnetic resonance imaging (MRI) of their dominant knee in 2003. Cartilage volume and bone marrow lesions (BMLs) were determined. All subjects also had retinal photographs taken from which retinal arteriolar and venular diameters were determined and summarized as the central retinal arteriolar equivalent (CRAE) and the central retinal venular equivalent (CRVE).

**Results:**

Retinal venular diameter was significantly wider in subjects with a BML compared with subjects without a BML (mean (SD) 214.2 (2.8) μm versus 207.5 (1.1) μm respectively independent of age, gender and BMI. A trend for decreased medial tibial cartilage with increasing CRAE was also observed (regression coefficient −2.70 μl, 95%CI-5.74, 0.5, p=0.08).

**Conclusion:**

These findings suggest that vascular pathology, indicative of inflammatory processes, is associated with early structural knee changes. The role of micro-vascular changes in the pathogenesis of OA warrants further investigation.

## Background

There is growing evidence that vascular disease might have a role in the pathogenesis of osteoarthritis (OA) [1]. People with OA have a higher prevalence of vascular disease and cardiovascular risk factors than those without OA [2]. More recently it was also found patients with OA are at higher risk of death from diabetes and cardiovascular disease compared to the general population [3]. Specific markers of vascular pathology including vessel wall thickness [4] and carotid artery calcification [5] have been also associated with the presence of generalised and hand OA respectively. While the study of vascular changes in hand OA [5] may not adequately represent the identical genetic, inflammatory and degenerative factors seen in knee OA. It still adds to the body of work suggesting vascular mechanism in OA. Whether the increase in vascular disease prevalence and mortality in OA populations is a result of the co-occurrence of cardiovascular disease and OA, which are both common in the older population, is due to OA treatments or to the common association with reduced physical activity and/or obesity is unclear [6].

One way to explore this is to examine asymptomatic people, prior to the onset of clinical knee OA. By using MRI it is possible to assess joint structure non-invasively. Structures such as cartilage volume and bone marrow lesions can be measured. Reduced cartilage volume is associated with increasing severity of OA and is linked to joint replacement [7], a clinically important outcome. Bone marrow lesions(BMLs) are associated with pain and progression of knee OA [8]. A further challenge is the difficulty of directly assessing bone microvasculature in healthy subjects. The retina provides a direct visualization of microvasculature in the body. Retinal microvasculature changes, such as arteriolar narrowing and venular widening, have been shown to be marker of generalized vascular pathology with reported associations with diabetes, hypertension, metabolic syndrome, atherosclerosis and inflammation and micro vascular disease (reviewed in [9]). Recently the relationships between retinal vascular calibre in patients with rheumatoid arthritis(RA) were explored [10]. Patients with RA were shown to have significantly wider retinal venular calibre compared to controls [10].

Therefore the aim of this study was to explore the cross-sectional relationship between changes in retinal microvasculature with BMLs and cartilage volume in a sample of asymptomatic community based adults.

## Methods

### Subjects

Subjects were recruited from the Melbourne Collaborative Cohort Study(MCCS). Subjects were excluded if they had OA, as defined by the American College of Rheumatology clinical criteria, current or past knee disease, a history of knee pain in the past five years at baseline lasting for > 24 hours; a previous knee injury requiring non-weight bearing treatment for > 24 hours or surgery (including arthroscopy); or a history of any arthritis diagnosed by a medical practitioner or contraindication to MRI. The study was approved by the Human Research Ethics Committee of The Cancer Council of Victoria and Monash University Standing Committee on Ethics in Research Involving Humans. All participants gave written informed consent.

#### Data collection

Participants completed a questionnaire that captured information on their demographics. Weight was measured to the nearest 0.1 kg (shoes, socks, and bulky clothing removed) using a single pair of electronic scales. Height was measured to the nearest 0.1 cm (shoes and socks removed) using a stadiometer. From these data, BMI (weight/height^2^ kg m^-2^) was calculated.

### Magnetic resonance imaging

A sagittal MRI of the dominant knee (defined as the lower-limb from which the subject stepped off when initiating gait) for each participant was performed between October 2003 and December 2004 as described on a 1.5-T whole body MR unit (Philips, Medical Systems, Eindhoven, the Netherlands) using a commercial transmit-receive extremity coil. The following sequences and parameters were used: fat suppressed, gradient recall acquisition in the steady state, three dimensional T1-weighted (58 msec/123 msec/55°, repetition time/echo time/ flip angle), 1 signal average, slice thickness 1.5 mm, field of view 16 cm and matrix 512 × 512. In addition a coronal T2-weighted fat-saturated acquisition, repetition time 2200 ms, echo time 20/80 ms, with a slice thickness of 3 mm, a 0.3 interslice gap, 1 excitation, a field of view of 11-12 cm, and a matrix of 256 × 128 pixels was also obtained [11].

#### Cartilage volume

Knee cartilage volume was determined by image processing on an independent workstation using the Osiris software (Digital Imaging Unit, University Hospital of Geneva, Switzerland). The volumes of individual cartilage plates (medial and lateral tibia) were measured from the total volume by manually drawing disarticulation contours around the cartilage boundaries on each section on a workstation as described from T1 images by 2 independent trained observers unpaired and blinded to subject identification The interclass correlation coefficients (ICC) were 0.94 for medial and 0.96 for lateral tibial cartilage volume measurements. The coefficients of intra-reader variation (CVs) for the medial and lateral tibial cartilage volume measures were 3.4% and 2.0% respectively [11].

#### Bone area

The cross-sectional areas of medial and lateral tibial plateaus were determined by means of image processing on an independent workstation using the software program OsiriX, by creating an isotropic volume from the input images, which were reformatted in the axial plane. Areas were directly measured from these axial images as previously described from T1 images by two assessors [11]. Using this technique, osteophytes, if present, are not included in the area of interest. The ICC (inter-class) were 0.97 for medial and 0.92 for lateral tibial plateau area measurements. The CVs for the medial and lateral tibial plateau bone areas were 2.3% and 2.4%, respectively [11].

#### Bone marrow lesions

BMLs were defined as areas of increased signal intensity adjacent to subcortical bone present in either the medial or lateral, distal femur or proximal tibia [12]. Two trained observers, who were blinded to patient characteristics, together assessed the presence of lesions for each subject from T2 coronal images [12]. The presence or absence of a BML was determined. The reproducibility for determination of the BML was assessed using 60 randomly selected knee MRIs (к value 0.88, P < 0.001).

### Measurement of retinal vascular calibre

**Figure 1:**
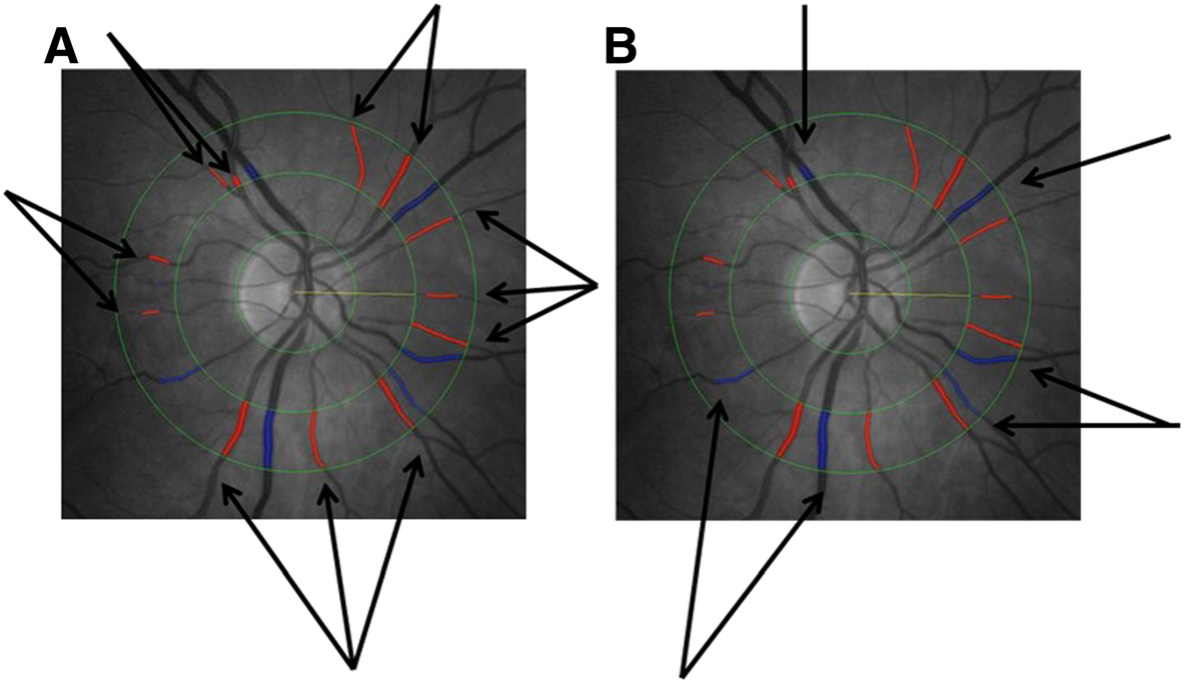
Retinal photograph with the arterioles and venules identified from which CRAE and CRVE are determined.

Non-mydriatic retinal photographs of both eyes, centred on the optic disc and the macula, were taken of participants using a digital retinal camera (Canon CR6-45NM; Canon, Lake Success, NY, USA). Retinal vascular calibre was measured using a computer-assisted vessel measurement system (University of Wisconsin, Madison, WI, USA) that uses microdensity to detect the vessel edge [13]. With the assistance of a trained grader, the program identifies all retinal vessels greater than 25 μm in diameter that completely pass through the region between 1/2 and 1 disc diameter from the optic disc margin (zone B) and identifies their edges using a pixel density histogram. The cross-sectional diameter of retinal arterioles and venules is measured repeatedly and summarized using formulae to obtain values representing the average arteriolar and venular caliber of that particular eye. The inter- and intra-grader agreement was examined in randomly selected 90 images. The inter- and intra-grader correlation coefficients for CRAE were 0.95 and 0.96 respectively. The inter- and intra-grader correlation coefficients for CRVE measurements were 0.98 and 0.99 respectively. Figure 1 provides a retinal photograph with the arterioles and venules identified from which CRAE and CRVE are determined. Because retinal calibre measures were highly correlated between eyes, vascular calibre measurements of the right eye were used in all analyses.

### Statistical analysis

Descriptive statistics for the characteristics of the study subjects were tabulated. All variables were assessed for normality. Retinal measures, cartilage volume and bone area were normally distributed. BMLs were dichotomous. The general linear model was used to compare retinal measures between those with and those without a BML. The estimated marginal mean was adjusted for age, gender and body mass index (model 1) and also either CRAE or CRVE (model 2). The relationship between retinal vasculature and cartilage volume was assessed by linear regression. The potential confounder’s age, gender, BMI and respective bone area (model 1) and also BMLs (yes v. No) (model 2) were included in multivariate analyses. A p value of less than 0.05 (two-tailed) was regarded as statistically significant. All analyses were performed using the SPSS statistical package (standard version 14.0, SPSS, Chicago, IL, USA).

## Results

Retinal photographs were available for 287(96%) participants. Study participants had a mean age of 58(SD5.5) years and BMI of 25.9(4.3) kg/m^2^. There were 186 women(61%). The mean CRAE and CRVE were 144.2(13.5) μm and 208.4(17.8) μm, respectively. Two participants(0.7%) had diabetes. Forty two(14%) people had a BML in their knee. People with a BML were not significantly older than those who did not have a BML (p= 0.22). The mean cartilage volume area at baseline was 1700 μl (SD 528) for the medial and 2042 μl (SD631) for the lateral compartment.

The mean vessel diameters for those with a BML and for those without a BML are presented in Table 1. No significant difference in retinal arteriolar diameter was observed between those with and those without a BML. In contrast, venular diameter was significantly wider for subjects with a BML compared with subjects without a BML both before (mean 214.1(SE2.8) μm for those with a BML compared with 207.5(SE1.1) μm, p=0.03) and after adjustment with age, gender and BMI (mean 214.2(SE2.8) μm for those with a BML compared with 207.5 (SE1.1) μm for those without a BML. No significant differences in arteriole diameters were observed. Similar findings were observed when the two subjects with diabetes were excluded from the analyses (data not shown).

**Table 1:** Estimated marginal means for arteriolar or venular diameter for those with and those without a BML

	**Mean**			**Adjusted mean†**		
	**BML**	**No BML**	**P value†**	**BML**	**No BML**	**P value†**
	**Mean (SE)**	**Mean (SE)**		**Mean (SE)**	**Mean (SE)**	
Arteriolar Diameter	147.4(2.1)	143.7(0.8)	0.11	147.5(2.1)	143.9(0.84)	0.11
Venular Diameter	214.1(2.8)	207.5(1.1)	**0.03**	214.2(2.8)	207.5(1.1)	**0.03**

†Estimated marginal mean, adjusting for age, gender, body mass index,

‡ Estimated marginal mean, adjusting for age, gender, body mass index, and either CRAE or CRVE.

The relationship between vessel diameter and medial and lateral tibial cartilage volume is presented in Table 2. Increasing arteriolar diameter was significantly associated with less medial tibial cartilage volume (regression coefficient −5.6 μl (95%CI-10.1, -1.1) change in cartilage volume per μm change in CRAE p=0.02) in univariate analyses. After adjustment the association remained the same but was no longer significant (regression coefficient −2.65 μl (95%CI −5.67, 0.38) p=0.09). Additional adjustment with BML presence did not change the association. No significant associations between arteriolar diameter and lateral tibial cartilage volume were observed. Similarly we did not observe any significant associations between venular diameter and either medial or lateral tibial cartilage volume. When the 2 diabetic subjects were excluded no significant differences were observed (data not shown).

**Table 2:** Cross-sectional relationships between vessel diameters and tibial cartilage volume

	**Univariate Analysis Regression Coefficient (95% CI)**	**P Value**	**Multivariate Analysis Regression Coefficient (95% CI)**†	**P Value**	**Multivariate Analysis Regression Coefficient (95% CI)**‡	**P Value**
*Medial Tibial Cartilage volume*						
Arteriolar Diameter	−5.6(−10.1, -1.1)	**0.02**	−2.65(−5.67, 0.38)	0.09	−2.70(−5.74, 0.5)	*0*.*08*
Venular Diameter	0.55(−2.97, 4.1)	0.76	−0.38(−2.67, 1.90)	0.74	−0.42(−2.73, 1.89)	0.72
*Lateral Tibial Cartilage volume*						
Arteriolar Diameter	−3.9 ( −9.55, 1.83)	0.18	0.63 ( −2.82, 4.12)	0.7	0.48(−3.02, 3.99)	0.79
Venular Diameter	2.14(−2.2, 6.5)	0.34	1.47 ( −1.15, 4.1)	0.27	1.34(−1.30, 3.98)	0.32

†adjusted for age, gender, BMI and respective bone area.

‡adjusted for age, gender, BMI, respective bone area and BMLs (yes v. No).

## Discussion

In our cross-sectional study of asymptomatic community based adults, mean retinal venular diameter was significantly wider in participants with a BML. A trend for reduced cartilage volume within the medial compartment with increasing arteriolar diameter was also observed.

In our study we found that those with BMLs had wider a CRVE. Wider venular calibre has been linked to diabetes, measures of arthrosclerosis, obesity and hypertriglyceridemia [9]. While the mechanisms linking the relationships between these conditions and increased venular diameter are unknown, there is some evidence to suggest that inflammatory processes or endothelial dysfunction underlie these relationships. Increased venular diameter has been associated with systemic markers of inflammation, such as C-reactive protein [14,15]. Venous occlusion and stasis may result in increased intra-osseous pressure and ischemia. Such changes have been reported in studies of bone marrow oedema in both human and animal models of OA [1,16,17]. It is well recognised that BMLs assessed on MRI may represent areas of osteonecrosis, oedema, trabecular abnormalities and bony remodeling [18] and there is evidence that they may be the consequence of episodes of ischemia and inflammation [19]. The findings of this study support a role for vascular changes possibly mediated by inflammatory factors. It is well known that the medial compartment is more susceptible to damage, most likely due to the increased loading through that compartment [20]. Our findings in the medial rather than the lateral compartment suggest a combination of systemic effects with local biomechanical effects.

The relationship between vascular markers and cartilage has not been previously explored. However, there is some evidence of a relationship with joint replacement, a marker of end stage OA [21]. Among women of the AGES-Reykjavik study, increased aortic calcium was associated with joint replacement (JR) due to OA. A trend towards increased atherosclerosis and JR was also observed [21]. In this study, we showed a trend for increasing arteriolar diameter and reduced medial, but not lateral, tibial cartilage volume. Cartilage is avascular receiving nutrients and oxygen from the underlying bone with more than 50% of the glucose, oxygen and water requirements of cartilage being provided by perfusion from the subchondral vessels [22]. Dilation of arterioles has been associated with markers of inflammation including higher leukocytes counts and higher erythrocyte sedimentation rate [15]. It is possible that inflammatory processes may affect the subchondral vessels resulting in the impaired the supply of nutrients to the overlying cartilage plate and subsequent cartilage loss. There is also evidence that during the degenerative process there is neovascularisation of the deep regions of articular cartilage [23]. It is possible that once early vascularisation of cartilage occurs inflammatory processes may influence cartilage directly. This has the potential to affect the structural integrity of cartilage and in the context of local biomechanical factors influence the progression of disease. Further longitudinal research will be required to assess this.

This study had a number of limitations. We have not examined vasculature in the bones directly. Our finding suggests that systemic factors are associated with knee changes cross-sectionally. It is possible that there are local factors affecting the vasculature that we have not been able to measure, further work will be required to assess this. The association of arterioles with cartilage volume did not reach significance, however given this was an asymptomatic population without evidence of clinical disease, and that a number of factors have been associated with cartilage volume cross-sectionally, it is not surprising that we did not detect a significant association between retinal vessel calibre and cartilage volume above the stronger risk factors. We did not assess femoral cartilage. A major limitation in measuring femoral cartilage volume is that the femoral cartilage articulates with the medial and lateral tibial cartilage and the patellar cartilage. There is no clear anatomical hallmark to separate the subregions of femoral cartilage that form part of each of these compartments so non-anatomical rules have been used to measure the femoral cartilage in different subregions. In addition tibial cartilage volume correlates with femoral cartilage volume in the same compartment [24] and grade of radiographic k****nee OA [25]. It is for these reasons that we have measured the tibial cartilage volume. This study was cross-sectional, therefore we cannot infer causality, larger longitudinal studies will be required. Finally due to the modest number of BMLs in our cohort, larger studies are needed which include examination of BML volume.

## Conclusion

We found that retinal venular diameter was wider in participants with a BML. A trend for reduced cartilage volume within the medial compartment with increasing arteriolar diameter was also observed. These findings were in asymptomatic community based adults who were free of clinical knee OA. These findings suggest a novel hypothesis that micro vascular pathology may be important in the pathogenesis of knee OA and warrants further work.

### Key messages

These findings suggest that vascular pathology is associated with early structural knee changes. The role of micro-vascular changes in the pathogenesis of OA warrants further investigation.

## Competing interest

The authors declared that they have no competing interest.

## Authors’ contributions

FC, AW, GG and English were involved in study design and inception. MLD-T, AW, FC were involved in subject recruitment, data collection, statistical analyses and interpretations. RK, TW and LH were involved in the retinal measures. All authors were involved in manuscript preparation and revision.

## Pre-publication history

The pre-publication history for this paper can be accessed here:

http://www.biomedcentral.com/1471-2474/13/255/prepub
